# Supplementary material for: Yiai Fuzheng decoction inhibits triple-negative breast cancer by remodeling the immune microenvironment
Source: Front Immunol. 2025 Sep 30;16:1615631. doi: 10.3389/fimmu.2025.1615631 (PMC12518410; doi:10.3389/fimmu.2025.1615631)
Supplement: Supplementary file 2 [file DataSheet1.zip › Supplementary File 2/Treatment/C_vs_H/07_Pathway_Analysis/Enrichment_Analysis/smpdb_pathway/Enrichment_Barplot.pdf]

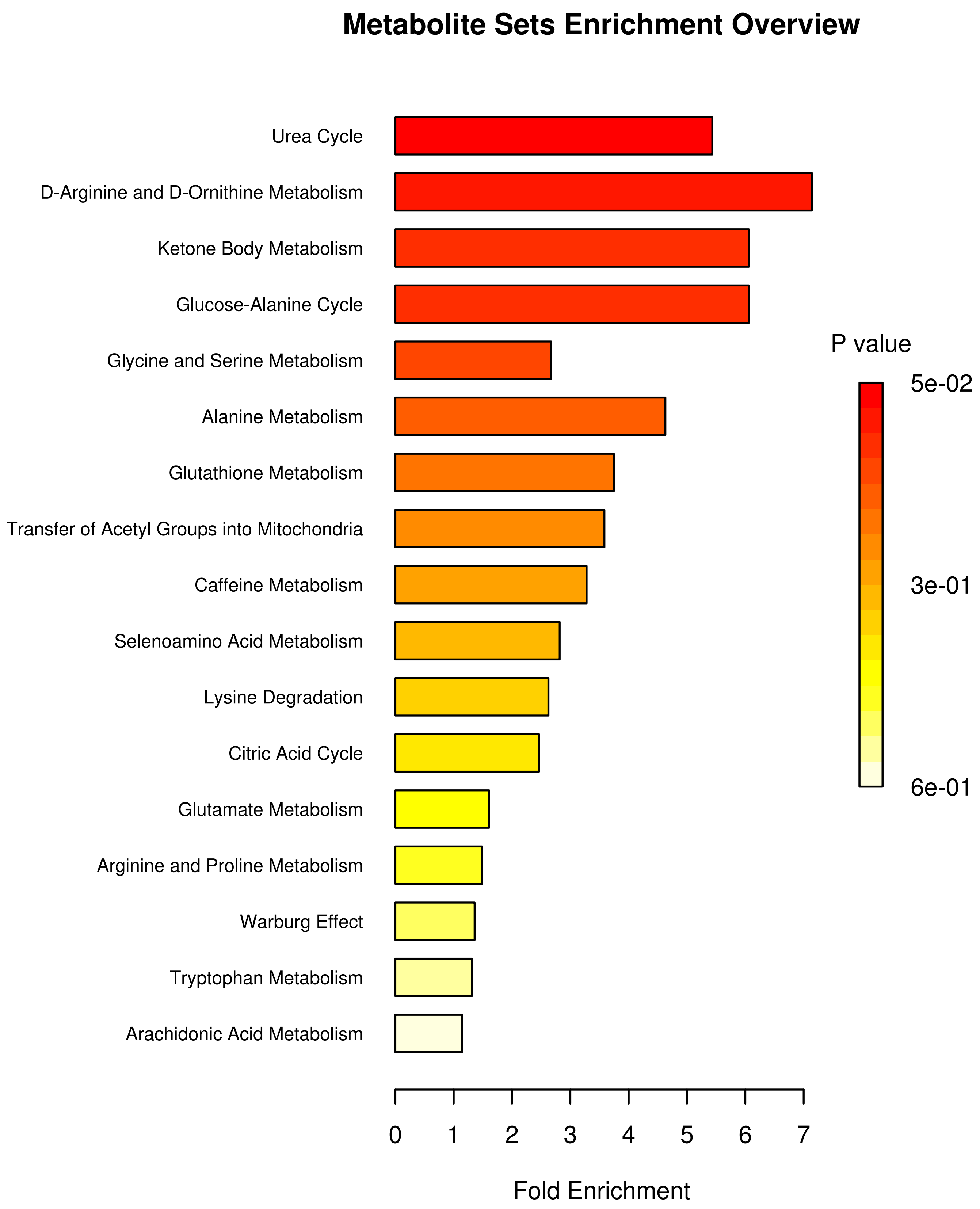

Metabolite Sets Enrichment Overview
Urea Cycle
D-Arginine and D-Ornithine Metabolism
Ketone Body Metabolism
Glucose-Alanine Cycle
Glycine and Serine Metabolism
Alanine Metabolism
Glutathione Metabolism
Transfer of Acetyl Groups into Mitochondria
Caffeine Metabolism
Selenoamino Acid Metabolism
Lysine Degradation
Citric Acid Cycle
Glutamate Metabolism
Arginine and Proline Metabolism
Warburg Effect
Tryptophan Metabolism
Arachidonic Acid Metabolism
0
1
2
3
4
5
6
7
Fold Enrichment
P value
5e-02
3e-01
6e-01
